# Are you a spontaneous traveler? Effect of sensation seeking on tourist planfulness in the mobile era

**DOI:** 10.3389/fpsyg.2022.968548

**Published:** 2022-08-10

**Authors:** Qiuyun Li, Hong Xu, Yubei Hu

**Affiliations:** ^1^Business School, Tianjin University of Finance and Economics, Tianjin, China; ^2^College of Tourism and Service Management, Nankai University, Tianjin, China; ^3^Warwick Manufacturing Group, University of Warwick, Coventry, United Kingdom

**Keywords:** sensation seeking, tourist planfulness, travel risk perception, smartphone usage, optimal level of stimulation theory

## Abstract

Drawn upon optimum stimulation level theory, and in view of the impact of mobile terminal usage on tourist decision-making, the present study aims to investigate how personality (i.e., sensation seeking) influences tourist trip planning behavior (i.e., tourist planfulness) in the mobile era. A sample of 344 respondents in China completed measures of sensation seeking, travel risk perception, smartphone usage, as well as tourist planfulness. Results indicated that sensation seeking was negatively associated with tourist planfulness and travel risk perception partially mediated this association. Besides, both the direct effect of sensation seeking on tourist planfulness and the indirect effect of travel risk perception were moderated by smartphone usage, in that these effects were stronger for tourists with a high-level of smartphone usage than those with low-level smartphone usage. This study can significantly advance existing research on tourist behavior from the perspective of personality and reconfiguring our traditional understanding on tourist decision-making in the mobile era. Our study may also provide indicative support for theoretical perspective that information technology is changing customer behavior.

## Introduction

The perspective of personality theories is one of the important approaches yet to be adequately explored to understand tourist behavior (e.g., Lee and Tseng, 2015; Masiero and Qiu, 2018; Talwar et al., 2022). As novelty seeking is one of the major travel motivations (Cohen, 1972; Crompton, 1979; Caber and Albayrak, 2016), researchers attempt to understand tourists’ behavior by borrowing the sensation seeking concept which implies that individuals vary in their preferred optimal level of stimulation (e.g., Walters et al., 2018; Zeng and He, 2019). Up to now, sensation seeking has attracted growing academic attention for decades and has been used to explain and predict tourist decision-making outcomes such as destination choice (Lee and Crompton, 1992; Masiero and Qiu, 2018), vacation preference (Wahlers and Etzel, 1985), or activity participation (Weber, 2001). In general, high sensation seekers prefer to choose exciting, adventurous, challenging, and diversified destination experience and activities, whereas the reverse is true for low sensation seekers. However, how personality influences trip planning, the tourist decision-making process leading to these choices remain in its infancy (Tan and Tang, 2013; Jani et al., 2014).

As travel is a special form of risk consumption involving intangibility, heterogeneity, and inseparability, and trip planning (e.g., information search, air ticket and hotel reservations, and tourist itinerary) are undertaken as a risk reduction strategy (Stewart and Vogt, 1999). For those whose aversion to risk leads them to careful considerations and detailed advance arrangements, planning takes on great importance (Roehl and Fesenmaier, 1992). Empirical research has confirmed that sensation seeking is negatively correlated with perceived risk (Rosenbloom, 2003; Zhang et al., 2016). We speculate that sensation seekers behave differently on trip planning from low sensation seekers. Whereas the former intentionally avoid preplans or make a loose plan to enjoy the spontaneous travel experience, the latter tend to collect as much destination information as possible before departure to work out a detailed travel plan. Therefore, we speculate that travelers varying in the amount of preplanning of their travel might be related to sensation seeking, and risk perception mediates the relationship.

Additionally, information and communication technologies (ICTs) have significantly revolutionized trip planning behavior (Gretzel et al., 2006; Xiang et al., 2015), and mobile ICTs further deepen this impact (e.g., Wang et al., 2012; Lamsfus et al., 2015). Mobile terminals represented by the smartphone prolong the online information search phase to the journey, enabling travelers to modify travel plans based on real-time location-based information or directly delay some decisions until en route (Fesenmaier and Jeng, 2000; Kramer et al., 2007; Wang et al., 2014). In the days before mobile terminals, a lengthy period of tourists’ decision-making spanned during the pre-trip stage at home. Tourists are becoming less planfulness or spontaneous in the mobile era as smartphones are considered ideal in supporting immediate and unreflective decisions en route (Hwang, 2010). The context of the mobile ICTs lends a new perspective on trip planning behavior. It is reasonable to assume that tourist decision-making processes have also evolved (McCabe et al., 2016; Liu et al., 2022), and it is time to investigate the new trends in the behavior change. Within this context, the purpose of this study is to investigate the underlying mechanism of trip planning behavior from the perspective of the combined effect of sensation seeking, travel risk perception, and mobile information technology usage.

## Literature review and hypothesis development

Travel provides an opportunity to make many choices, e.g., destination, transportation, lodging, dining, and the very nature of travel requires tourists to make some decisions to go on the trip in advance of the departure. Therefore, as a specific type of tourist decision-making process that includes information search, booking, and even paying (Hyde, 2008), trip planning is considered one of the central behavioral aspects of travel. Existent research on trip planning behavior has been measured from different perspectives, e.g., the lead time tourists take to make their plans (Money and Crotts, 2003), serendipitous or organized travel style (Huang et al., 2016), tourists’ autonomy in trip planning (FernÁNdez-Herrero et al., 2018), or tourists’ perceptions of information sources (Alvarez and Asugman, 2006). For example, Money and Crotts (2003) proposed that tourists from high risk-avoidance societies would tend to plan their trips longer in advance and make their reservations earlier than tourists in medium uncertainty avoidance cultures. Moreover, according to tourists’ perceptions of online and offline information sources for planning their vacations, Alvarez and Asugman (2006) identified spontaneous explorers and risk-averse planners who hold different attitudes toward planning beforehand. Based on the above discussions, it seems that trip planning behavior measurement to date is skimble-scamble. In the present study, we use the term “planfulness” from action style theory (Frese et al., 1987) to describe the tourist’s tendency to elaborate a detailed trip plan proactively for a particular travel.

Because goals and plans guide our actions, Frese et al. (1987) described interindividual differences in goal orientation and planfulness as action styles. They believed that action styles are neither traits nor aspects of temperament nor abilities. They are propensities to act, which are represented cognitively as certain general learned heuristics for how to act. And they are teachable to a certain degree because it is possible to tell people to plan carefully and they will most probably abide. In addition, action styles are bidirectional as an action style should be modified depending on the specifics of a situation (Frese et al., 1987). Thereinto, the term “planfulness” refers to the general tendency to plan in detail, develop backup plans in case a plan goes wrong, and persist in pursuing plans (von Papstein and Frese, 1988). Planfulness is predicted to be associated with lower initial decision-making, as a more planful individual spends more time working out the details of their intended actions (Tucker and Warr, 1996). Based on action style theory, we define tourist planfulness as the degree to which people make detailed travel plans about how to proceed in advance of action.

We propose that sensation seeking will negatively associate with tourist planfulness based on optimal level of stimulation theory (OLST). OLST believes that individuals have a preferred level of stimulation in their lives (Hebb and Thompson, 1954), and Zuckerman (1979) coined the concept sensation seeking accordingly. He defined sensation seeking as “a trait defined by the need for varied, novel and complex sensations and experiences and the willingness to take physical and social risks for the sake of such experience” (Zuckerman, 1979). His discussions of sensation seeking in the field of tourism indicated that low sensation seekers like to plan their trips very carefully or have it planned for them because they like the comfortable familiarity of their usual environment. By contrast, high sensation seekers do not worry much about booking all of their reservations in advance and may change their itinerary on impulse as they travel (Zuckerman, 1994). This logic thus suggests that sensation seeking is in line with tourist role described by Cohen’s (1972) and Plog’s (2002) typology. Specifically, Cohen’s novelty seeking tourists (explorers and drifters) and Plog’s venture tourists prefer to travel freely or spontaneously, whereas, organized or independent mass tourists and allocentric tourists are likely to pre-plan much of their trip or buy packaged tours directly. By integrating these theoretical propositions, this study assumes that sensation seeking is an important personality trait that predicts tourist planfulness. That is, sensation seekers tend to be less planful regarding upcoming travels to enjoy the spontaneous, unexpected, and novelty travel experience.

As an important risk reduction strategy, trip planning is conceptually related to travel risk perception. When tourists perceive a risky situation, they may gather more information (Michalko, 2004), alter their travel plans by shifting from traveling alone to traveling in groups or travel individually to package tours (Adam, 2015) or even find a different destination (Decrop, 2010). It suggests that travel risk perception is a strong predictor of tourist planfulness. We propose that the higher the tourists perceive travel risk, the more time they tend to spend in information search and elaborating specific travel itineraries in advance. Sensation seeking and risk perception do not only affect individual behavior separately. Previous research established risk perception as mediators that link sensation seeking with risky behavior such as adolescent cigarette smoking (Doran et al., 2011), alcohol use (Urbán et al., 2008), and reckless driving (Rundmo and Iversen, 2004) and suggested that sensation seekers have a higher target risk level and perceive the level of risk to be lower than sensation avoiders. This lower risk perception is in part due to sensation seekers having a much higher level of optimism than sensation avoiders (Heino et al., 1996) and they consider taking risks as a way to gain sensation. However, those findings were mostly based on health/safety content and Zhang et al. (2016) found that the mediation models of the sensation seeking on risky behavior through risk perception vary in health/safety, recreational and social, ethical domains. Therefore, it is necessary to identify whether this mechanism regarding planning behavior exists in the tourism research filed.

Fuchs (2013) found that sensation seeking and travel risk perceptions are negatively correlated, while Lepp and Gibson (2008) demonstrated that sensation seeking was not related to perceptions of risk. To date, it is, however, not clear how and to what extent sensation seeking and risk perception are related in terms of tourism (Karl and Schmude, 2017). Although the debate and controversy remain, further research is needed to continue to improve our understanding of the complex nature of this issue. Given the literature on sensation seekers who are not attracted by risk but are more willing to take risks in order to gain sensation as a reward, these individuals are more inclined to travel spontaneously to enjoy the novelty travel experience. This indicates that sensation seeking may also decrease tourist planfulness indirectly through lower perceptions of risk. Hence, we proposed that travel risk perception is positively related to tourist planfulness, and it also mediates the relationship between sensation seeking and tourist planfulness.

Tourism is an important application field of mobile ICTs (Buhalis, 2020; Lv et al., 2020), and the number of individuals using social medias on smartphone to plan their trips is increasing. Firstly, the smartphone offers a convenient channel that tourists can use it at any time to search for information, check availability and book or pay, navigate to destinations, and so forth (Lamsfus et al., 2015; Liu et al., 2022; Moon and An, 2022). In view of that smartphone usage can extend tourist decision-making process phase until en route, tourist’s tendency to elaborate a detailed trip plan proactively will be declining accordingly, especially for high sensation seekers who are far more willing to try the new applications of smartphone under new circumstances (i.e., the unusual environment of travel), thus to be more spontaneous and take risks. As evidenced in past studies, using smartphone in travel planning may thus help tame tourist worries about travel itinerary by mitigating the uncertainty and anxiety of tourists (Pana et al., 2021; Goo et al., 2022). Whereas, the low sensation seekers would instead doing something following the beaten track, prefer to organize their trips seamlessly before departure and think this is reliable and safe. As such, we expect that the higher the level of smartphone usage, the stronger the negative effect of sensation seeking on tourist planfulness. Secondly, Internet-specific features help consumers reduce their purchase risk and establish expectations (FernÁNdez-Herrero et al., 2018; Yang et al., 2021). Consumers could acquire clear signals of service quality of various tourism service suppliers by reading the review comments on social media such as Dianping, Yelp, Ctrip, or Expedia. In view of the aforementioned hypothesis that travel risk perception mediates the relationship between sensation seeking and tourist planfulness, we assume that this indirect effect depends on smartphone usage level, that is, the mediated relationship will be stronger for tourist with high-level smartphone usage than with low-level smartphone usage.

**FIGURE 1:**
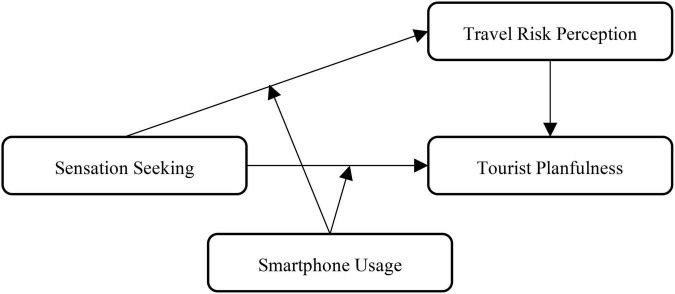
The proposed model.

In sum, the present study aims at testing a moderated mediation model, which integrates five assumptions in one model: (a) sensation seeking negatively affects tourist planfulness (H1); (b) travel risk perception positively affects tourist planfulness (H2); (c) travel risk perception mediates the relationship between sensation seeking and tourist planfulness (H3); (d) smartphone usage level moderates the relationship between sensation seeking and tourist planfulness (H4); and (e) the mediation model also depends on the level of smartphone usage (H5). Figure 1 depicts the conceptual model and hypotheses of this research.

## Materials and methods

### Participants and procedure

Participants were recruited through SOJUMP (a popular online questionnaire survey platform in China), and they were asked to recall their last non-business travel experience and answer questions based on their memory. Sojump’s records indicated that 418 members responded to our survey. And forty-five participants failed the attention check question and twenty-nine participants filled out the questionnaire in less than 120 s. These participants were excluded from all analyses, leaving a final sample of 344 valid responses. The valid returned rate was 82.30%. The participants were 56.40% female, and most were between 18 and 39 years old (85.17%) and got a bachelor’s degree or above (90.70%).

### Measures

Sensation seeking was assessed with the Steinberg et al. (2008) version of the six-item (e.g., “I like doing things just for the thrill of it;” “I sometimes like to do things that are a little frightening.”) on a five-point Likert scale, 1 (not at all true of me) to 5 (totally true of me). The Cronbach’s alpha for this study was 0.917.

Travel risk perception was measured with the 11-item scale (e.g., “I concerned that the trip to the destination would result in physical danger, injury or sickness;” “I concerned in encountering strange food while on vacation”) suggested by Li et al. (2015) on a five-point Likert scale, 1 (strongly disagree) to 5 (strongly agree). These items were developed on the desk research of Roehl and Fesenmaier (1992); Lepp and Gibson (2003), and Lepp et al. (2011). The reliability estimate of α = 0.89.

Combined with travel contextual factors, the survey was adapted from the general planfulness scale (Frese et al., 1987). The authors developed the initial set of items and then modified them based on five experts’ advice. Afterward, a pilot study used seven items (e.g., “I planned the trip far in advance before departure;” “I made very detailed plans regarding the trip before departure.”) on a five-point Likert scale [1 (strongly disagree) to 5 (strongly agree)] with a sample of 67 was conducted to validate the adapted version and had a Cronbach’s alpha of 0.846, which is far above acceptable standards. Cronbach’s α of the scale in the present study was 0.871.

Smartphone usage in the present study refers to tourists’ proficiency in using smartphones to assist their travels. It was measured by a summed score based on four items we self-developed based on the desk research of Wang et al. (2014). The regulated four items were “I could skillfully use the smartphone to do information search while traveling,” “I could skillfully use the smartphone to purchase tourism products (e.g., book airline ticket, make hotel reservation, buy entrance tickets) while traveling,” “I could skillfully use the smartphone to conduct global position system (GPS) navigation while traveling,” and “I could skillfully use the smartphone to make an itinerary while traveling.” Response values to each item ranged from 1 (strongly disagree) to 5 (strongly agree). The internal consistency was 0.909.

We controlled for trip characteristics [spatial distance from tourism market to destinations, trip duration, travel mode, first or repeat visitors of the destination, travel purpose, number of traveling companions, and the old (>60) or children (<14) in the traveling companions (yes or no)], as they have been found to influence travel risk perception (e.g., Roehl and Fesenmaier, 1992; Lepp and Gibson, 2003). We also controlled for participants past travel experience and sociodemographic characteristics (gender, age, income, and education) to make more robust inferences regarding the theoretical model that cannot simply be attributed to a common third factor.

Content validity refers to the extent to which the scale could capture the essence of the latent variable that has to be measured. Since we derived most of the scale items in our questionnaire through a comprehensive study of previous relevant literature and mature instruments, the content validity of our instrument was established.

### Data analysis

Common method deviation test and discriminant validity test were conducted firstly, and then the bivariate relationship was assessed by computing the Pearson *r* coefficients using SPSS 19.0. Furthermore, regression analysis and Models of PROCESS SPSS macro (Hayes, 2013) were conducted to test our hypotheses.

## Results

### Common method deviation test

In response to the deviation of the common method caused by the self-report method used in the data collection, the data collection process was controlled (e.g., the survey was performed anonymously, and reverse problems were designed for some items) at first. Harman’s single-factor test was also used to check the common method biases. The results show that there were six factors of characteristic roots >1, among which the first factor explained the variation of 22.05%, <40%, indicating that the common method deviation is within the acceptable range.

### Discriminant validity test

Discriminant validity refers to how divergent the scores of a test are from other variables that assess different constructs (Sireci and Sukin, 2013). In order to establish discriminant validity, confirmatory factor analysis was performed. The results indicated that the four-factor model fit index was superior to other alternative models like three or two-factor models (*χ*^2^ = 1,276.1, *df* = 344, *χ*^2^/*df* = 3.710, *CFI* = 0.835, *IFI* = 0.836, *RMSEA* = 0.080). This means that the four variables in this study have good discriminant validity in terms of connotation and measurement, and are indeed four different constructs.

### Correlational analysis

To evaluate the relationships between variables preliminarily, we computed correlations. Table 1 shows the means, standard deviations, and correlation coefficients of study variables. As shown in the table, sensation seeking was significantly and negatively related to travel risk perception and tourist planfulness, while positively associated with smartphone usage. Tourist risk perception was significantly positively correlated with tourist planfulness, while significantly and negatively associated with smartphone usage. Smartphone usage had a significant negative correlation with the tourist planfulness.

**TABLE 1:** Means, standard deviations, and zero-order correlations among study variables.

Variables	*M*	*SD*	1	2	3	4
(1) Sensation seeking	2.98	0.98	1			
(2) Travel risk perception	2.51	0.80	−0.28**	1		
(3) Tourist planfulness	2.89	0.90	−0.28**	0.43**	1	
(4) Smartphone usage	4.17	0.81	0.15**	−0.24**	−0.19**	1

*p < 0.05; **p < 0.01.

### Mediation analyses

To examine the main effect (H1, H2) and mediation effect (H3), we performed a series of regression analyses suggested by Baron and Kenny (1986): (1) the independent variable (X; sensation seeking) predicts the dependent variable (Y; tourist planfulness); (2) the independent variable (X) predicts the mediator (M; travel risk perception); (3) the mediator (M) and the independent variable (X) predict the dependent variable (Y) with the effect of X on Y that becomes not significant or that decreases when controlling for M. All the trip characteristic and personal feature variables were added as control variables, and so were the following analysis.

As Table 2 showed the total effect of sensation seeking on tourist planfulness (Model 4) was statistically significant (β = −0.275, *p* < 0.01), thereby supporting Hypothesis 1. The effect of sensation seeking on travel risk perception (Model 2) was statistically significant (β = −0.304, *p* < 0.01), and the effect of travel risk perception on tourist planfulness (Model 5) was statistically significant (β = 0.352, *p* < 0.01). Hence, Hypothesis 2 was supported. Finally, the magnitude of the direct effect of sensation seeking on travel risk perception when controlling for the effect of travel risk perception (Model 6) has decreased (β = −0.185, *p* < 0.01) compared with the total effect of sensation seeking on tourist planfulness, suggesting a partial mediation. Namely, travel risk perception partially mediated the association between sensation seeking and tourist planfulness.

**TABLE 2:** Regression results for main effect and mediation effect.

Variables	Travel risk perception		Tourist planfulness		Tourist planfulness	
	Model 1	Model 2	Model 3	Model 4	Model 5	Model 6
Spatial distance	0.02	0.01	0.06	0.05	0.05	0.05
Length of stay	0.12*	0.13*	0.18**	0.19**	0.14*	0.15**
Travel mode	0.04	0.03	0.13*	0.13*	0.12*	0.12*
Ever been there	0.00	0.01	0.08	0.09	0.08	0.09
Travel purpose	0.09	0.12*	–0.02	0.00	–0.06	–0.03
Number of people in the travel partners	0.08	0.07	0.06	0.05	0.03	0.03
Children or aged people in the travel partners	–0.00	–0.02	–0.02	–0.04	–0.02	–0.03
Gender	–0.09	−0.14**	−0.11*	−0.16**	–0.08	−0.11*
Age	0.01	–0.04	0.11	0.06	0.10	0.07
Education	−0.17**	−0.17**	−0.18**	−0.18**	−0.12*	−0.13*
Monthly disposable income	0.00	0.02	–0.03	–0.02	–0.03	–0.02
Previous travel experience	−0.13*	−0.12*	–0.11	–0.10	–0.07	–0.06
Sensation seeking		−0.30**		−0.27**		−0.19**
Travel risk perception					0.35**	0.30**
*R* ^2^	0.09**	0.18**	0.17**	0.24**	0.29**	0.32**
△*R*^2^		0.09**		0.07*		0.03**

*p < 0.05; **p < 0.01.

Parameter estimates are standardized.

A modern approach using bootstrapping was bought forward and advocated to further infer the intervening variable effects (Hayes and Preacher, 2010). In the present study, we followed this procedure and employed bootstrapping analyses of the sampling distribution to test the indirect effect (H3). At first, we centered all continuous variables by standardizing to a mean of 0 and a standard deviation of 1 (Aiken et al., 1991). Then, for the parameter estimates of the mediating variable, we calculated 95% percentile confidence intervals (CIs) with *N* = 5,000 bootstrap re-samples using the SPSS macros Process Model 4. Bootstrapping analyses indicated that sensation seeking exerted an indirect effect (a*b = −0.090) on tourist planfulness through the intervention of travel risk perception (95% CI = −0.143 to −0.046). Furthermore, the effect of sensation seeking to tourist planfulness (*a* = −0.185, *p* < 0.01, 95% CI = −0.284 to −0.087) was still significant after controlling the mediation variable. That is, travel risk perception had partly intermediate function which sensation seeking affected tourist planfulness. Thus, Hypothesis 3 was supported.

### Moderation and moderated mediation analysis

**FIGURE 2:**
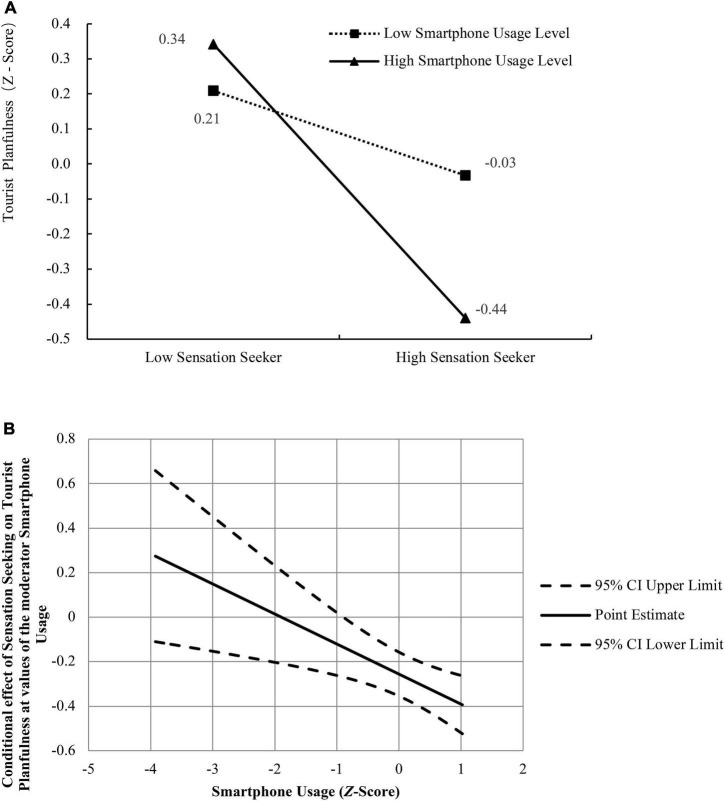
**(A)** Plot of the relationship between sensation seeking and tourist planfulness at two levels of smartphone usage. **(B)** Graphing of the conditional effect of sensation seeking on tourist planfulness at values of the moderator smartphone usage.

To test the moderation effect where smartphone usage moderates the relation between sensation seeking and tourist planfulness (Hypothesis 4), Model 1 of the SPSS macro Process was conducted with *N* = 5,000 bootstrap re-samples, and again, all continuous variables were standardized. The results revealed that sensation seeking had a significant effect on tourist planfulness (β = −0.256, *t* = −5.109, 95% CI = −0.354 to −0.157, *p* < 0.01) and the interaction between sensation seeking and travel risk perception was also significant and negative (β = −0.135, *t* = −2.876, 95% CI = −0.227 to −0.043, *p* < 0.01). Namely, smartphone usage moderated the association between sensation seeking and tourist planfulness. To better understand this moderating effect, the plot of the relation between sensation seeking and tourist planfulness at two levels of smartphone usage (1 *SD* below the mean and 1 *SD* above the mean) was described in Figure 2A (Aiken et al., 1991). As can be seen from Figure 2A, for individuals with high-level smartphone usage (1 *SD* above the mean), sensation seeking was strongly associated with tourist planfulness, while this association was weaker for individuals with low-level smartphone usage (1 *SD* below the mean). Specifically, the effect of sensation seeking on tourist planfulness was significant at high-level of smartphone usage (*b* = −0.391, *t* = −5.974, 95% CI = −0.520 to −0.262, *p* < 0.001), weak but not significant at low-level (*b* = −0.121, *t* = −1.685, 95% CI = −0.262 to 0.020, *p* = 0.093). The conditional effect of sensation seeking on tourist planfulness at values of the moderator smartphone usage was also graphed in Figure 2B.

**FIGURE 3:**
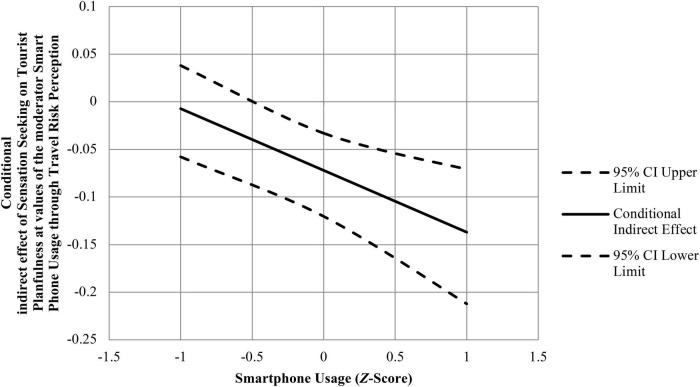
Graphing of the conditional indirect effect of sensation seeking on tourist planfulness at values of the moderator smartphone usage through travel risk perception.

We subsequently tested a moderated mediation model in which smartphone usage moderated the effect of sensation seeking on travel risk perception, and in which this interaction helped predict tourist planfulness. The PROCESS macro for Model 8 with *N* = 5,000 bootstrap re-samples showed a significant moderated mediation pattern (index of moderated mediation = −0.065, Boot *SE* = 0.021, 95% CI = −0.109 to −0.029). As can be seen from Table 3, the mediator variable model for predicting travel risk perception, sensation seeking was negatively correlated with travel risk perception (β = −0.26, *p* < 0.001), while the interaction of sensation seeking and smartphone usage was negatively correlated with travel risk perception (β = −0.24, *p* < 0.001); the dependent variable model for predicting tourist planfulness, sensation seeking was negatively correlated with tourist planfulness (β = −0.18, *p* < 0.001), while the interaction of sensation seeking and smartphone usage was negatively correlated with tourist planfulness, but it is not significantly (β = −0.07, *p* = 0.136). The conditional indirect effect analysis showed that the indirect effect of sensation seeking on tourist planfulness through travel risk perception was moderated by smartphone usage. For individuals with high-level smartphone usage (1 *SD* above the mean), there is a strong and significant indirect effect of travel risk perception (conditional indirect effect = −0.137, Boot *SE* = 0.036, 95% CI = −0.212 to −0.071). For individuals with low-level smartphone usage (1 *SD* below the mean), however, the indirect effect was much weaker and not significant (conditional indirect effect = −0.007, Boot *SE* = 0.024, 95% CI = −0.058 to 0.038). Thus, Hypothesis 6 was practically supported. The conditional indirect effect of sensation seeking on tourist planfulness at values of the moderator smartphone usage through travel risk perception is graphically displayed in Figure 3.

**TABLE 3:** Regression results for moderated mediation model.

Variables	Travel risk perception					Tourist planfulness					
	β	*SE*	*t*	95% CI		β	*SE*	*t*	95% CI	
Spatial distance	0.02	0.06	0.41	–0.09	0.14	0.06	0.06	1.00	–0.05	0.17
Length of stay	0.11*	0.06	1.98	0.00	0.23	0.15**	0.06	2.62	0.04	0.25
Travel mode	0.01	0.05	0.20	–0.09	0.11	0.12*	0.05	2.38	0.02	0.22
Ever been there	0.02	0.05	0.43	–0.08	0.13	0.10	0.05	1.89	0.00	0.19
Travel purpose	0.11*	0.05	2.19	0.01	0.21	–0.03	0.05	–0.63	–0.13	0.07
Number of people in the travel partners	0.06	0.06	1.15	–0.05	0.17	0.03	0.05	0.61	–0.07	0.14
Children or aged people in the travel partners	–0.05	0.14	–0.34	–0.32	0.22	–0.07	0.13	–0.54	–0.33	0.19
Gender	–0.18	0.10	–1.74	–0.38	0.02	−0.21*	0.10	–2.13	–0.40	–0.02
Age	–0.02	0.06	–0.30	–0.13	0.10	0.08	0.06	1.44	–0.03	0.19
Education	−0.17**	0.05	–3.14	–0.27	–0.06	−0.13**	0.05	–2.62	–0.24	–0.03
Monthly disposable income	0.00	0.06	0.01	–0.11	0.11	–0.03	0.05	–0.58	–0.14	0.07
Previous travel experience	–0.09	0.06	–1.67	–0.20	0.02	–0.06	0.05	–1.19	–0.17	0.04
Sensation seeking	−0.26**	0.05	–5.22	–0.36	–0.16	−0.18**	0.05	–3.66	–0.28	–0.09
Smartphone usage	−0.18**	0.05	–3.36	–0.28	–0.07	–0.02	0.05	–0.38	–0.12	0.08
Sensation seeking × smartphone usage	−0.24**	0.05	–5.01	–0.33	–0.14	–0.07	0.05	–1.50	–0.16	0.02
Risk perception						0.27**	0.05	5.19	0.17	0.38
*R*	0.50					0.57				
*R* ^2^	0.25					0.32				
*F*	7.39					9.64				

*p < 0.05; **p < 0.01.

Continuous variables was centered by standardizing to a mean of 0 and a standard deviation of 1.

## Discussion

Drawn upon optimum stimulation level theory and the tourist decision-making literature, we developed and tested a theoretical model linking sensation seeking with tourist trip planning behavior by investigating the underlying mechanisms as well as the boundary conditions. The results showed that sensation seeking was associated with tourist planfulness not only directly but also indirectly through the mediation of travel risk perception. And travel risk perception had a direct positive significant effect on tourist planfulness. Besides, the direct effect of sensation seeking on tourist planfulness and the indirect effect through travel risk perception were both moderated by smartphone usage, with these two effects being stronger for individuals with high-level smartphone usage. This study can significantly advance existing research on tourist behavior and reconfiguring our traditional understanding on tourist decision-making.

### Theoretical implications

Our study contributes to the tourist behavior literature by extending previous research in the following ways. First, our findings have important theoretical implications for tourist behavior research from the perspective of personality. Although sensation seeking has been previously identified as predictors of tourist decision-making outcomes such as destination choice, activity preference, there is little research available on the decision-making process leading to these choices (Tan and Tang, 2013). We have answered this call by hatching the concept of tourist planfulness to describe tourists’ trip planning behavior characteristics and investigating how personality (i.e., sensation seeking) influences it. Second, we also sought to explore how such effects come to be by identifying the mediating role of travel risk perception. By doing so, our findings support that the relationship between sensation seeking and tourist planfulness would be partially mediated by travel risk perception. This finding contributes to a better understanding of the role that risk perception might play between sensation seeking and leisure behavior since previous studies were mostly based on health/safety content (Zhang et al., 2016). Third, to our knowledge, this is the very first such investigation of these particular mechanisms within the context of mobile information technology.

In consideration of the potential impact of mobile ICTs on tourist behavior (Wang et al., 2014), our study goes one step further by uncovering that both the direct path from sensation seeking to tourist planfulness, and the indirect path through travel risk perception were much stronger for individuals who could skillfully use smartphones to do trip planning tasks such as information search, navigation, booking or paying. That is, if being able to use smartphones to do trip planning, sensation seekers tend to be less planful, and it also reduced the toursits’ risk perception, which could strengthen the negative effect of sensation seeking on tourist planfulness. On the other hand, among tourists who could not skillfully use smartphones assist their trips en route, sensation seekers may still tend to fully prepare their upcoming trips before departure, which could buffer the nagative relationship between sensation seeking and tourist planfulness. This pattern also suggests that the effect of mobile ICTs on tourist behavior deserves more attention, as they can help channel the influence of personality in such a mobile information time. These findings are consistent with the results indicating the impact of mobile terminals on tourist behavior (e.g., Xiang et al., 2015; Liu et al., 2022). They may also provide indicative support for theoretical perspective that the use of information technology is changing our behavior (Helbing, 2019).

### Practical inspirations

The present study had practical implications for the tourism industry. Travel and tourism businesses are supposed to recognize the trip planning nuances among different personality groups and develop strategies that specifically target high and low sensation seekers. For example, for low sensation seekers, marketing resources should be assigned to their trip planning stage at home, as they feel safe and comfortable after having obtained substantial information related to the destination and plan a trip in detail, while, on-site marketing efforts target high sensation seeking tourists. As the impact of today’s mobile ICTs on tourist behavior is deepened day by day, tourism enterprises and destination management organizations should re-recognize the decision-making process, which is becoming more spontaneous and dynamic. Various strategies of mobile ICTs (e.g., real-time communication, navigation services, mobile payment) and online/offline relationship management programs can be used to cater to the on-the-go tourists (Lamsfus et al., 2015).

### Limitations and future directions

The present study has certain limitations that should be addressed by future research. First, one may argue that the adequacy and representativeness of our sample might not be justified. While according to the sample-to-variable ratio, 15–20 observations per independent variable are recommended (Hair et al., 2018), so the sample size was not small nor is it considered large as we included 16 independent variables in the multiple regressions. In addition, most of the participants are under 40 years old and highly educated. This was mainly caused by the sampled population on SOJUMP platform as its age distribution and occupational structure data showed that the registered participants are composed mostly of young and educated people. Caution should be exercised in extrapolating the findings to the aged and not highly educated population, and future work is needed to examine the effect of sensation seeking on tourist planfulness in the mobile era with a more diverse and representative sample. Second, due to the correlational cross-sectional nature of the study design, any definitive conclusions regarding causality cannot be made. Although trip characteristics, individual demographic, and past travel experience variables were controlled as covariates, alternative explanations to the observed associations cannot be ruled out without experimental manipulation or temporally tracking. Longitudinal research designs and laboratory or quasi-experiments should be encouraged on how personality influences tourist behavior under the mobile information technology in subsequent research. Third, more research is needed to identify other antecedents of trip planning behavior from the personality perspective, such as impulsive personality, risk-taking propensity, and the mechanism of how ICTs exerting their influence. Despite these limitations, these results should encourage academics and practitioners to take into account the effects of smart phone usage on tourist decision-making process in the mobile era.

## Data availability statement

The original contributions presented in this study are included in the article/supplementary material. Further inquiries can be directed to the corresponding author.

## Author contributions

QL: theoretical building of the manuscript and original draft preparation. HX: data collection, reviewing, and editing. YH: analysis of data, writing, and editing. All authors contributed to the article and approved the submitted version.

## References

[B1] Adam I. (2015). Backpackers’ risk perceptions and risk reduction strategies in Ghana. *Tour. Manag.* 49 99–108. 10.1016/j.tourman.2015.02.016

[B2] Aiken L. S. West S. G. Reno R. R. (1991). *Multiple regression: Testing and interpreting interactions.* Newbury Park, CA: Sage Publications.

[B3] Alvarez M. Asugman G. (2006). Explorers versus planners: A study of Turkish tourists. *Ann. Tour. Res.* 33 319–338.

[B4] Baron R. M. Kenny D. A. (1986). The moderator-mediator variable distinction in social psychological research: Conceptual, strategic, and statistical considerations. *J. Pers. Soc. Psychol.* 51 1173–1182. 10.1037/0022-3514.51.6.1173 3806354

[B5] Buhalis D. (2020). Technology in tourism-from information communication technologies to eTourism and smart tourism towards ambient intelligence tourism: A perspective article. *Tour. Rev.* 75 267–272. 10.1108/TR-06-2019-0258

[B6] Caber M. Albayrak T. (2016). Push or pull? Identifying rock climbing tourists’ motivations. *Tour. Manag.* 55 74–84. 10.1016/j.tourman.2016.02.003

[B7] Crompton J. L. (1979). Motivations for pleasure vacation. *Ann. Tour. Res.* 6 408–424.

[B8] Cohen E. (1972). Toward a sociology of international tourism. *Soc. Res.* 39 164–182.

[B9] Decrop A. (2010). Destination choice sets: An inductive longitudinal approach. *Ann. Tour. Res.* 37 93–115. 10.1016/j.annals.2009.08.002

[B10] Doran N. Sanders P. E. Bekman N. M. Worley M. J. Monreal T. K. McGee E. (2011). Mediating influences of negative affect and risk perception on the relationship between sensation seeking and adolescent cigarette smoking. *Nicotine Tobacco Res.* 13 457–465. 10.1093/ntr/ntr025 21436297 PMC3103719

[B11] FernÁNdez-Herrero M. HernÁNdez-Maestro R. M. GonzÁLez-Benito Ó (2018). Autonomy in trip planning and overall satisfaction. *J. Travel Tour. Mark.* 35 119–129. 10.1080/10548408.2017.1350250

[B12] Fesenmaier D. R. Jeng J. M. (2000). Assessing structure in the pleasure trip planning process. *Tour. Anal.* 5 13–27.

[B13] Frese M. Stewart J. Hannover B. (1987). Goal orientation and planfulness: Action styles as personality concepts. *J. Pers. Soc. Psychol.* 52:1182. 10.1037/0022-3514.52.6.1182

[B14] Fuchs G. (2013). Low versus high sensation-seeking tourists: A study of backpackers’ experience risk perception. *Int. J. Tour. Res.* 15 81–92. 10.1002/jtr.878

[B15] Goo J. Huang C. D. Yoo C. W. Koo C. (2022). Smart tourism technologies’ ambidexterity: Balancing tourist’s worries and novelty seeking for travel satisfaction. *Inf. Syst. Front.* 1–20. 10.1007/s10796-021-10233-6 35103046 PMC8791698

[B16] Gretzel U. Fesenmaier D. R. Formica S. O’Leary J. T. (2006). Searching for the future: Challenges faced by destination marketing organizations. *J. Travel Res.* 45 116–126. 10.1177/0047287506291598

[B17] Hair J. F. Black W. C. Babin B. J. Anderson R. E. (2018). *Multivariate data analysis*, 8th Edn. London: Cengage Learning.

[B18] Hayes A. F. (2013). *Introduction to mediation, moderation, and conditional process analysis: A regression-based approach.* New York, NY: Guilford Press.

[B19] Hayes A. F. Preacher K. J. (2010). Quantifying and testing indirect effects in simple mediation models when the constituent paths are nonlinear. *Multiv. Behav. Res.* 45 627–660. 10.1080/00273171.2010.498290 26735713

[B20] Hebb D. O. Thompson W. R. (1954). “The social significance of animal studies,” in *Handbook of social psychology*, ed. Lindzey G. (Reading, MA: Addison-Wesley), 551–552.

[B21] Heino A. van der Molen H. H. Wilde G. J. S. (1996). Risk perception, risk taking, accident involvement and the need for stimulation. *Saf. Sci.* 22 35–48. 10.1016/0925-7535(96)00004-5

[B22] Helbing D. (eds.) (2019). “Societal, economic, ethical and legal challenges of the digital revolution: From big data to deep learning, artificial intelligence, and manipulative technologies,” in *Towards Digital Enlightenment*, (Cham: Springer), 47–72. 10.1007/978-3-319-90869-4_6

[B23] Huang W.-J. Hallo J. C. Norman W. C. McGehee N. G. McGee J. Goetcheus C. (2016). “To plan or ot to plan:” serendipitous vs. organized travel. *Tour. Travel Res. Assoc. Adv. Tour. Res. Glob.* 39. Available online at: http://scholarworks.umass.edu/tra/2010/Oral/39

[B24] Hwang Y. H. (2010). A theory of unplanned travel decisions: Implications for modeling on-the-go travelers. *Inf. Technol. Tour.* 12 283–296. 10.3727/109830511X12978702284516 30089248

[B25] Hyde K. F. (2008). Information processing and touring planning theory. *Ann. Tour. Res.* 35 712–731. 10.1016/j.canrad.2009.06.026 19766526

[B26] Jani D. Jang J. H. Hwang Y. H. (2014). Big five factors of personality and tourists’ Internet search behavior. *Asia Pac. J. Tour. Res.* 19 600–615. 10.1080/10941665.2013.773922

[B27] Karl M. Schmude J. (2017). Understanding the role of risk (perception) in destination choice: A literature review and synthesis. *Tour. Int. Interdiscip. J.* 65 138–155.

[B28] Kramer R. Modsching M. Hagen K. Gretzel U. (2007). “Behavioural impacts of mobile tour guides,” in *Information and communication technologies in tourism*, eds Sigala M. Mich L. Murphy J. (Vienna: Springer), 109–118. 10.1007/978-3-211-69566-1_11

[B29] Lamsfus C. Wang D. Alzua-Sorzabal A. Xiang Z. (2015). Going mobile: Defining context for on-the-go travelers. *J. Travel Res.* 54 691–701. 10.1177/0047287514538839

[B30] Lee T. H. Crompton J. (1992). Measuring novelty seeking in tourism. *Ann. Tour. Res.* 19 732–751. 10.3390/ijerph17010122 31877974 PMC6981675

[B31] Lee T. H. Tseng C. H. (2015). How personality and risk-taking attitude affect the behavior of adventure recreationists. *Tour. Geogr.* 17 307–331.

[B32] Lepp A. Gibson H. (2003). Tourist roles, perceived risk and international tourism. *Ann. Tour. Res.* 30 606–624. 10.1016/S0160-7383(03)00024-0

[B33] Lepp A. Gibson H. (2008). Sensation seeking and tourism: Tourist role, perception of risk and destination choice. *Tour. Manag.* 29 740–750. 10.1016/j.tourman.2007.08.002

[B34] Lepp A. Gibson H. Lane C. (2011). Image and perceived risk: A study of Uganda and its official tourism website. *Tour. Manag.* 32 675–684. 10.1016/j.tourman.2010.05.024

[B35] Li J. Pearce P. L. Wu B. Morrison A. M. (2015). The impact of smog on risk perception and satisfaction of international and domestic tourists in Beijing. *Tour. Trib.* 30 48–59.

[B36] Liu X. Wang D. Gretzel U. (2022). On-site decision-making in smartphone-mediated contexts. *Tour. Manag.* 88:104424. 10.1016/j.tourman.2021.104424

[B37] Lv X. Li C. McCabe S. (2020). Expanding theory of tourists’ destination loyalty: The role of sensory impressions. *Tour. Manag.* 77:104026. 10.1016/j.tourman.2019.104026

[B38] Masiero L. Qiu R. T. (2018). Modeling reference experience in destination choice. *Ann. Tour. Res.* 72 58–74. 10.1016/j.annals.2018.06.004

[B39] McCabe S. Li C. Chen Z. (2016). Time for a radical reappraisal of tourist decision making? Toward a new conceptual model. *J. Travel Res.* 55 3–15. 10.1177/0047287515592973

[B40] Michalko G. (2004). Tourism eclipsed by crime: The vulnerability of foreign tourists in Hungary. *J. Travel Tour. Mark.* 15 159–172. 10.1300/J073v15n02_09

[B41] Money R. B. Crotts J. C. (2003). The effect of uncertainty avoidance on information search, planning, and purchases of international travel vacations. *Tour. Manag.* 24 191–202. 10.1016/S0261-5177(02)00057-2

[B42] Moon J. W. An Y. (2022). Scale construction and validation of uses and gratification motivations for smartphone use by tourists: A multilevel approach. *Tour. Hosp.* 3 100–113. 10.3390/tourhosp3010007

[B43] Pana B. Lin M. S. Liang Y. Akyildiz A. Park S. Y. (2021). Social, ethical, and moral issues in smart tourism development in destinations. *J. Smart Tour.* 1 9–17. 10.52255/smarttourism.2021.1.1.3

[B44] Plog S. (2002). The power of pyschographics and the conturesomeness. *J. Travel Res.* 40 244–251. 10.1177/004728750204000302

[B45] Roehl W. S. Fesenmaier D. R. (1992). Risk perceptions and pleasure travel: An exploratory analysis. *J. Travel Res.* 30 17–26. 10.1177/004728759203000403

[B46] Rosenbloom T. (2003). Risk evaluation and risky behavior of high and low sensation seekers. *Soc. Behav. Pers. Int. J.* 31 375–386. 10.2224/sbp.2003.31.4.375

[B47] Rundmo T. Iversen H. (2004). Risk perception and driving behaviour among adolescents in two Norwegian counties before and after a traffic safety campaign. *Saf. Sci.* 42 1–21. 10.1016/S0925-7535(02)00047-4

[B48] Sireci S. G. Sukin T. (2013). “Test validity,” in *APA handbook of testing and assessment in psychology: Vol 1. Test theory and testing and assessment in industrial and organizational psychology*, eds Geisinger K. F. Bracken B. A. Carlson J. F. Hansen J.-I. C. Kuncel N. R. Reise S. P. (Washington, DC: American Psychological Association), 61–84. 10.1037/14047-004

[B49] Steinberg L. Albert D. Cauffman E. Banich M. Graham S. Woolard J. (2008). Age differences in sensation seeking and impulsivity as indexed by behavior and self-report: Evidence for a dual systems model. *Dev. Psychol.* 44:1764. 10.1037/a0012955 18999337

[B50] Stewart S. I. Vogt C. A. (1999). A case-based approach to understanding vacation planning. *Leis. Sci.* 21 79–95. 10.1080/014904099273165

[B51] Talwar S. Srivastava S. Sakashita M. Islam N. Dhir A. (2022). Personality and travel intentions during and after the COVID-19 pandemic: An artificial neural network (ANN) approach. *J. Bus. Res.* 142 400–411. 10.1016/j.jbusres.2021.12.002 34924646 PMC8669890

[B52] Tan W. K. Tang C. Y. (2013). Does personality predict tourism information search and feedback behaviour? *Curr. Issues Tour.* 16 388–406. 10.1080/13683500.2013.766155

[B53] Tucker P. Warr P. (1996). Intelligence, elementary cognitive components, and cognitive styles as predictors of complex task performance. *Pers. Individ. Dif.* 21 91–102. 10.1016/0191-8869(96)00032-3

[B54] Urbán R. Kökönyei G. Demetrovics Z. (2008). Alcohol outcome expectancies and drinking motives mediate the association between sensation seeking and alcohol use among adolescents. *Addict. Behav.* 33 1344–1352. 10.1016/j.addbeh.2008.06.006 18619739

[B55] von Papstein P. Frese M. (1988). “Transferring skills from training to the actual work situation: The role of task application knowledge, action styles and job decision latitude,” in *Proceedings of the SIGCHI conference on Human factors in computing systems. Conference on Human Factors in Computing Systems*, Vol. 15 ed. O’Hare J. J. (New York, NY: Association for Computing Machinery), 55–60. 10.1145/57167.57176

[B56] Wahlers R. G. Etzel M. J. (1985). Vacation preference as a manifestation of optimal stimulation and lifestyle experience. *J. Leis. Res.* 17 283–295. 10.1080/00222216.1985.11969638

[B57] Walters G. Wallin A. Hartley N. (2018). The threat of terrorism and tourist choice behavior. *J. Travel Res.* 58 370–382. 10.1177/0047287518755503

[B58] Wang D. Park S. Fesenmaier D. R. (2012). The role of smartphones in mediating the touristic experience. *J. Travel Res.* 51 371–387. 10.1177/0047287511426341

[B59] Wang D. Xiang Z. Fesenmaier D. R. (2014). Adapting to the mobile world: A model of smartphone use. *Ann. Tour. Res.* 48 11–26. 10.1016/j.annals.2014.04.008

[B60] Weber K. (2001). Outdoor adventure tourism: A review of research approaches. *Ann. Tour. Res.* 28 360–377. 10.1016/S0160-7383(00)00051-7

[B61] Xiang Z. Wang D. O’Leary J. T. Fesenmaier D. R. (2015). Adapting to the internet: Trends in travelers’ use of the web for trip planning. *J. Travel Res.* 54 511–527. 10.1177/0047287514522883

[B62] Yang Y. Liu Y. Lv X. Ai J. Li Y. (2021). Anthropomorphism and customers’ willingness to use artificial intelligence service agents. *J. Hosp. Mark. Manag.* 31 1–23. 10.1080/19368623.2021.1926037

[B63] Zeng B. He Y. (2019). Factors influencing Chinese tourist flow in Japan–a grounded theory approach. *Asia Pac. J. Tour. Res.* 24 56–69. 10.1080/10941665.2018.1541185

[B64] Zhang L. Zhang C. Shang L. (2016). Sensation-seeking and domain-specific risk-taking behavior among adolescents: Risk perceptions and expected benefits as mediators. *Pers. Individ. Dif.* 101 299–305. 10.1007/s10899-020-09992-9 33389431

[B65] Zuckerman M. (1979). *Sensation seeking: Beyond the optimal level of arousal.* Hillsdale, NJ: Lawrence Erlbaum Associates.

[B66] Zuckerman M. (1994). *Behavioral expressions and biosocial bases of sensation seeking.* Cambridge, MA: Cambridge University Press.

