# Corrigendum: Are you a spontaneous traveler? Effect of sensation seeking on tourist planfulness in the mobile era

**DOI:** 10.3389/fpsyg.2022.1081316

**Published:** 2022-12-13

**Authors:** Qiuyun Li, Hong Xu, Yubei Hu

**Affiliations:** ^1^Business School, Tianjin University of Finance and Economics, Tianjin, China; ^2^College of Tourism and Service Management, Nankai University, Tianjin, China; ^3^Warwick Manufacturing Group, University of Warwick, Coventry, United Kingdom

**Keywords:** sensation seeking, tourist planfulness, travel risk perception, smartphone usage, optimal level of stimulation theory

In the published article, there was an error in the **Funding** statement. Two funding projects were erroneously excluded. The corrected **Funding** statement appears below.

